# Differential Diagnosis of Akinetic Mutism and Disorder of Consciousness Using Diffusion Tensor Tractography: A Case Report

**DOI:** 10.3389/fnhum.2022.778347

**Published:** 2022-02-25

**Authors:** Dong Hyun Byun, Sung Ho Jang

**Affiliations:** Department of Physical Medicine and Rehabilitation, College of Medicine, Yeungnam University, Daegu, South Korea

**Keywords:** diffusion tensor tractography (DTT), akinetic mutism, disorder of consciousness (DOC), prefronto-caudate tract, prefronto-thalamic tract

## Abstract

This paper presents a case in whom a differential diagnosis of akinetic mutism with a disorder of consciousness was made using diffusion tensor tractography (DTT). A 69-year-old female patient was diagnosed with subarachnoid hemorrhage, intraventricular hemorrhage, and intracerebral hemorrhage produced by the subarachnoid hemorrhage. She exhibited impaired consciousness with a Coma Recovery Scale-Revised score of 13 until 1 month after onset. Her impaired consciousness recovered slowly to a normal state according to the Coma Recovery Scale-Revised (23 points: full score) at 7 weeks after onset. On the other hand, she exhibited the typical clinical features of akinetic mutism (no spontaneous movement [akinesia] or speech [mutism]). On the DTT performed at 1-month, the upper, and lower dorsal ascending reticular activating systems, which are related to a disorder of consciousness, showed an almost normal state. In contrast, the prefronto-caudate and prefronto-thalamic tracts, which are related to akinetic mutism, showed severe injuries. These DTT results suggested that the patient's main clinical features were not a disorder of consciousness but akinetic mutism. Therefore, DTT for the ascending reticular activating system, and the prefronto-caudate and prefronto-thalamic tracts could provide additional evidence for a differential diagnosis of DOC and AM at the early stages of stroke.

## Introduction

A differential diagnosis of akinetic mutism (AM) and disorder of consciousness (DOC) can be clinically difficult at the early stages of a brain injury. AM is a rare neurological disorder of impaired initiation and motivation for behavior (Arnts et al., 2020). The representative clinical features of AM are a lack of voluntary movement (akinesia) and absence of speech (mutism), but eye-opening and spontaneous or environmentally induced visual tracking are maintained (Arnts et al., 2020). Bilateral disruption of the fronto-subcortical circuit has been suggested as an important pathophysiological mechanism of AM (Mega and Cohenour, 1997; Nagaratnam et al., 2004; Marin and Wilkosz, 2005; Jang and Kwon, 2017). In particular, an injury to the cortico-striatal-pallidal-thalamic circuit is considered the most plausible pathophysiological mechanism (Mega and Cohenour, 1997; Nagaratnam et al., 2004; Marin and Wilkosz, 2005; Jang and Kwon, 2017). However, precise reconstruction of the fronto-subcortical circuit in a live human brain has been impossible. The introduction of diffusion tensor tractography (DTT), which is derived from diffusion tensor imaging, enables the estimation and visualization of some neural tracts of the fronto-subcortical circuit, including the prefronto-caudate and prefronto-thalamic (mediodorsal nucleus) tracts (Behrens et al., 2007; Leh et al., 2007; Jang and Yeo, 2014; Jang and Kwon, 2017). As a result, several DTT-based studies have reported that AM is related to injuries of the above two neural tracts, particularly the prefronto-caudate tract (Jang and Kwon, 2017; Jang et al., 2017a,b, 2018). On the other hand, DTT also allows a reconstruction of the ascending reticular activating system (ARAS), which is an important neural network for controlling consciousness (Jang et al., 2019a,b). Thus, this study hypothesized that reconstruction of the neural tracts related to AM and DOC using DTT could be useful in a differential diagnosis of AM and DOC.

This case study describes a case of AM, which was confirmed from DOC by the clinical features and DTT.

## Case Description

A 69-year-old female patient was diagnosed with subarachnoid hemorrhage caused by an aneurysm rupture in the anterior communicating artery and intraventricular hemorrhage, and intracerebral hemorrhage in both basal forebrains produced by a subarachnoid hemorrhage (Fisher grade 4) (Fisher et al., 1980). She underwent coiling and extraventricular drainage through the right prefrontal lobe on the day of onset and ventriculoperitoneal shunt for hydrocephalus 5 days after onset at the neurosurgery department of a general hospital. Approximately 1 month after onset, she was transferred to the rehabilitation department of a University hospital. The patient exhibited impaired consciousness (obedient to simple commands, such as eye closing, eye tracking to visual stimuli, and head turning to the side of auditory stimuli), with a Coma Recovery Scale-Revised (full score: 23; a higher score indicates higher consciousness) score of 13 (auditory function, 3 [reproducible movement to command]; visual function, 4 [object localization: reaching]; motor function, 2 [flexion withdrawal]; verbal function, 1 [oral reflexive movement]; communication, 1 [non-functional: intentional]; and arousal, and 2 [eye opening without stimulation] (Giacino et al., 2004).

**Figure 1:**
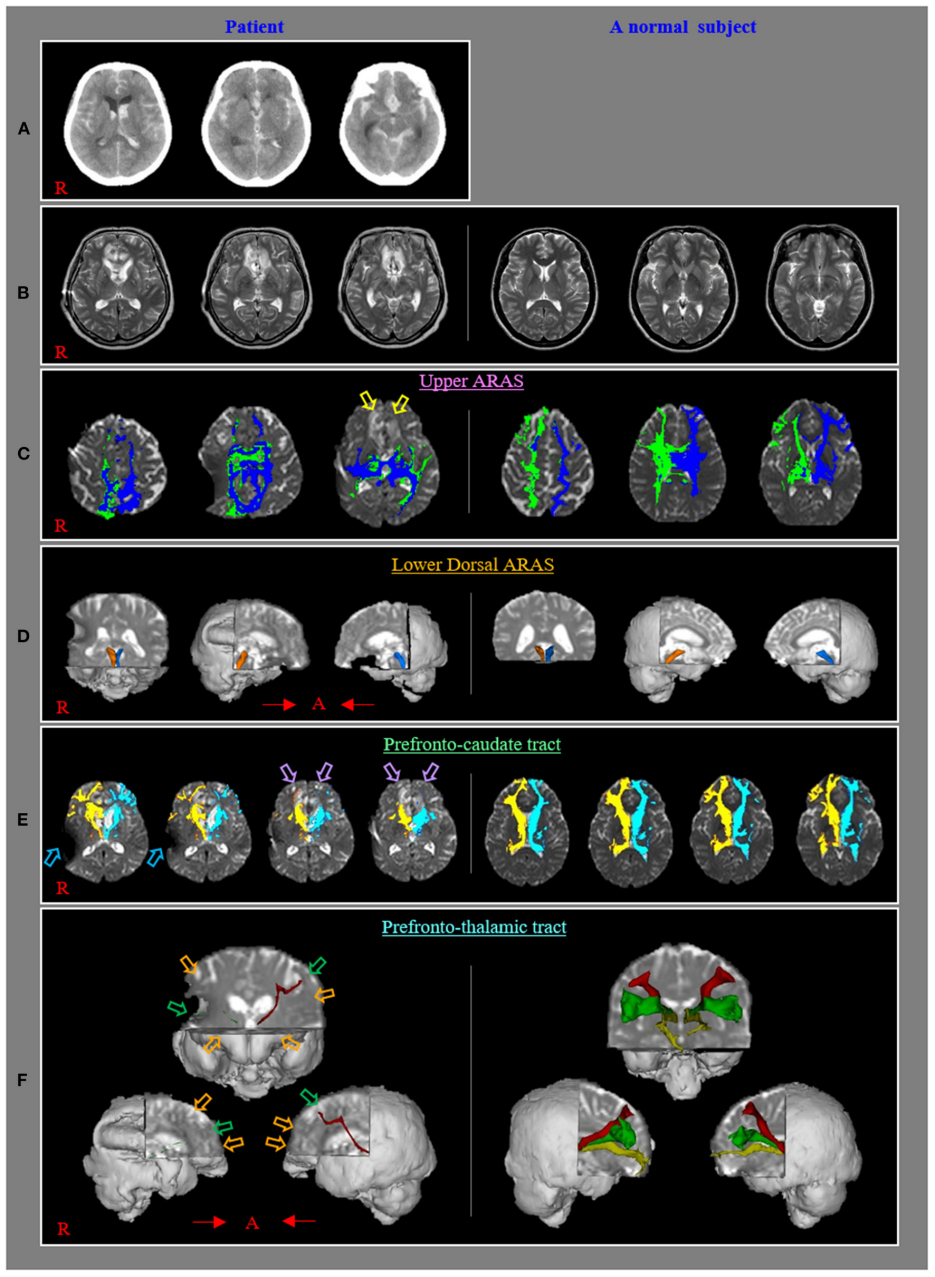
**(A)** Brain CT images at onset reveal subarachnoid hemorrhage, intraventricular hemorrhage, and intracerebral hemorrhage in both basal forebrains. **(B)** T2-weighted brain MR images at 1 month after onset show leulomalactic lesions in both forebrains. **(C)** The upper ascending reticular activating system (ARAS) shows almost normal configurations in both hemispheres except for decreased neural connectivities to both basal forebrains (yellow arrows) compared with those of a normal control subject (50-year-old female). **(D)** The lower dorsal ARAS reveals almost normal configurations in both hemispheres compared with those of a normal control subject (62-year-old female). **(E)** The neural connectivity of the caudate nucleus to the prefrontal cortex decreased in both hemispheres (violet arrows) compared to those of a normal control subject (50-year-old female) (sky-blue arrows: artifact due to ventriculoperitoneal shunt, which was performed through the right parietal approach). **(F)** All prefronto-thalamic tracts are not reconstructed (orange arrows) except for the right ventrolateral and left dorsolateral tracts, which show severe thinning (green arrows) compared to those of a normal control subject (60-year-old female).

Brain magnetic resonance images taken 1 month after onset revealed leukomalactic lesions in both basal forebrains (Figure 1A). Her impaired consciousness recovered slowly to a normal state as Coma Recovery Scale-Revised (23 points) (auditory function, 4 [consistent movement to commend]; visual function, 5 [object recognition]; motor function, 6 [functional object use]; verbal function, 3 [intelligible verbalization]; communication, 3 [oriented]; and arousal, and 3 [attention) at 7 weeks after onset (Giacino et al., 2004). However, she showed no spontaneous movement or speech and remained in a lying position all day with no spontaneous activity. However, she could execute movements and speak according to the clinician's order with some preservation of awareness. The patient's daughter provided signed, informed consent, and the institutional review board approved the study protocol.

The diffusion tensor imaging data were acquired 1 month after onset using a 1.5 T Philips Gyroscan Intera (Philips, Ltd., Best, Netherlands) with a six-channel head coil and single-shot echo-planar imaging. For each of the 32 non-collinear diffusion-sensitizing gradients, contiguous slices parallel to the anterior commissure–posterior commissure line were acquired. The imaging parameters were as follows: acquisition matrix = 96 × 96, reconstructed to matrix = 192 × 192 matrix, field of view = 240 mm × 240 mm, TR = 10,398 ms, TE = 72 ms, parallel imaging reduction factor (SENSE factor) = 2, EPI factor = 59, b = 1,000 s/mm^2^, NEX = 1, and slice thickness = 2.5 mm. The diffusion-weighted imaging data were analyzed using tools within the Oxford Center for Functional Magnetic Resonance Imaging of the Brain (FMRIB) Software Library (FSL; www.fmrib.ox.ac.uk/fsl). Affine multi-scale two-dimensional registration was used to correct the head motion effects and image distortion due to eddy currents. Fiber tracking was performed using a probabilistic tractography method based on a multifiber model and was applied using the tractography routines implemented in FMRIB Diffusion software (5,000 streamline samples, 0.5 mm step lengths, curvature thresholds = 0.2; corresponding to a minimum angle of 80°). All regions of interest (ROIs) were applied manually based on the previous studies and atlas (Daube, 1986; Morel et al., 1997; Afifi and Bergman, 2005; Johansen-Berg et al., 2005; Kringelbach, 2005; Petrides, 2005; Brodmann and Gary, 2006; Leh et al., 2007; Morel, 2007; Klein et al., 2010; Yeo et al., 2013; Jang and Yeo, 2014; Jang et al., 2014; Mendoza and Eblen-Zajjur, 2019). Two portions of the ARAS were reconstructed by selecting the fibers passing through the following regions of interest (ROIs): lower dorsal ARAS (seed ROI, the pontine reticular formation [RF], target ROI, thalamic intralaminar nucleus [ILN] at the level of the inter-commissural plane between the anterior and posterior commissures), and the upper ARAS (neural connectivity of the ILN to the cerebral cortex) (Daube, 1986; Morel et al., 1997; Afifi and Bergman, 2005; Morel, 2007; Yeo et al., 2013; Jang et al., 2014). Based on 5,000 samples generated from the seed voxel, the results for contact were visualized at a minimum threshold of two for the lower dorsal ARAS and 10 for the neural connectivity of the ILN (upper ARAS). For the connectivity of the caudate nucleus (CN) to the prefrontal cortex (PFC), the seed region of interest (ROI) was placed on the caudate nucleus, which was isolated by the adjacent structures (medial boundary: the lateral ventricle, lateral boundary: the anterior limb of the internal capsule) (Leh et al., 2007; Yeo et al., 2013; Mendoza and Eblen-Zajjur, 2019). To reconstruct the prefronto-thalamic tracts (Johansen-Berg et al., 2005; Kringelbach, 2005; Petrides, 2005; Brodmann and Gary, 2006; Klein et al., 2010; Jang and Yeo, 2014), a seed ROI was placed on the known anatomical location of the mediodorsal nucleus of the thalamus on the coronal image (Johansen-Berg et al., 2005; Klein et al., 2010; Jang and Yeo, 2014). Each target ROI was as follows: (1) dorsolateral PFC as Brodmann areas (BAs) 8, 9, and 46 on the coronal image; (2) ventrolateral PFC as BAs 44, 45, and 47 on the coronal image; and (3) orbitofrontal cortex as BAs 47, 11, and 13 on the axial image (Kringelbach, 2005; Petrides, 2005; Brodmann and Gary, 2006; Klein et al., 2010; Jang and Yeo, 2014). The prefronto-thalamic tracts were determined by selecting the fibers passing through the seed and each target ROI.

The upper and lower dorsal ARAS showed almost normal configurations in both hemispheres except for decreased neural connectivities to both basal forebrains (Figures 1B,C). The neural connectivity of the CN to the prefrontal cortex was decreased in both hemispheres (Figure 1D). None of the prefronto-thalamic tracts were reconstructed except for the right ventrolateral and left dorsolateral tracts, which showed severe thinning (Figures 1E,F).

## Discussion

This patient showed a DOC until she was admitted to the rehabilitation department 1 month after onset. She also revealed the typical clinical features of AM (complete absence of spontaneous behavior [akinesia] and speech [mutism]) when her consciousness had recovered to a normal state 7 weeks after onset (Marin and Wilkosz, 2005). As a result, her main clinical features were not DOC but AM. In detail, it appeared that she had combined clinical features of AM (main) and DOC (minor) between 1 month and 7 weeks after onset because she could not execute movements and speak according to the clinician's order. Subsequently, at 7 weeks when her consciousness had recovered to a normal state, she presented typical clinical features of AM (akinesia and mutism) without clinical features of DOC because she could execute movements and speak according to the clinician's order. In addition, on 1-month DTT, the prefronto-caudate and prefronto-thalamic tracts showed severe injuries whereas the ARAS revealed mild injuries. Thus, when she was transferred to our hospital at 1 month after onset, we could assume that her main clinical features were not DOC but AM based on 1-month DTT findings.

Severe injury of the fronto-subcortical circuit (particularly, the prefronto-caudate, and prefronto-thalamic tracts) has been suggested as the pathophysiological mechanism of AM (Mega and Cohenour, 1997; Nagaratnam et al., 2004; Jang and Kwon, 2017; Jang et al., 2017a,b, 2018). The upper and lower dorsal ARAS showed an almost normal state in this patient, whereas the prefronto-caudate and prefronto-thalamic tracts revealed severe injuries. These DTT results appeared to coincide with the patient's main clinical features of AM. This study had some limitations. First, the whole fronto-subcortical circuit could not be reconstructed except for the prefronto-caudate and prefronto-thalamic tracts because a reconstruction method for the whole fronto-subcortical circuit has not been developed. Second, the results of DTT can be false positives or negatives due to crossing fibers and partial volume effects (Yamada et al., 2009). Third, follow up DTTs from the acute stage could provide better evidences. However, we could not scan the diffusion tensor imaging at the acute stage because she was transferred from other hospital.

In conclusion, a differential diagnosis of AK with DOC was made in this patient using the clinical features and DTT findings. The results suggest that DTT for the ARAS, prefronto-caudate tract, and prefronto-thalamic tract could be additional evidence for a differential diagnosis of DOC and AM at the early stages of a stroke. On the other hand, further studies will be needed to apply these DTT methods for other brain pathologies, such as hypoxic-ischemic brain injury, traumatic brain injury, and global ischemia.

## Data Availability Statement

The raw data supporting the conclusions of this article will be made available by the authors, without undue reservation.

## Ethics Statement

The studies involving human participants were reviewed and approved by Yeungnam University Hospital. The patients/participants provided their written informed consent to participate in this study. Written informed consent was obtained from the individual(s) for the publication of any potentially identifiable images or data included in this article.

## Author Contributions

DB: study concept, design, and critical revision of manuscript for intellectual content. SJ: study concept and design, manuscript development, writing, funding, and critical revision of manuscript for intellectual content. All authors contributed to the article and approved the submitted version.

## Funding

This work was supported by the National Research Foundation of Korea (NRF) grant funded by the Korean Government (MSIP) (No. 2021R1A2B5B01001386).

## Conflict of Interest

The authors declare that the research was conducted in the absence of any commercial or financial relationships that could be construed as a potential conflict of interest.

## Publisher's Note

All claims expressed in this article are solely those of the authors and do not necessarily represent those of their affiliated organizations, or those of the publisher, the editors and the reviewers. Any product that may be evaluated in this article, or claim that may be made by its manufacturer, is not guaranteed or endorsed by the publisher.
